# Trabecular Bone Microarchitecture Improvement Is Associated With Skeletal Nerve Increase Following Aerobic Exercise Training in Middle-Aged Mice

**DOI:** 10.3389/fphys.2021.800301

**Published:** 2022-02-22

**Authors:** Seungyong Lee, Yun-A Shin, Jinkyung Cho, Dong-ho Park, Changsun Kim

**Affiliations:** ^1^Department of Physiology, College of Graduate Studies, Midwestern University, Glendale, AZ, United States; ^2^Department of Exercise Prescription and Rehabilitation, College of Sports Science, Dankook University, Cheonan, South Korea; ^3^Department of Sport Science, Korea Institute of Sport Science, Seoul, South Korea; ^4^Department of Kinesiology, Inha University, Incheon, South Korea; ^5^Department of Biomedical Science, Program in Biomedical Science and Engineering, Inha University, Incheon, South Korea; ^6^Department of Physical Education, Dongduk Women’s University, Seoul, South Korea

**Keywords:** aerobic exercise training, BMD, BMC, trabecular bone microarchitecture, skeletal nerves

## Abstract

Advancing age is associated with bone loss and an increased risk of osteoporosis. Exercise training improves bone metabolism and peripheral nerve regeneration, and may play a critical role in osteogenesis and increase in skeletal nerve fiber density. In this study, the potential positive role of aerobic exercise training in bone metabolism and skeletal nerve regeneration was comprehensively evaluated in 14-month-old male C57BL/6 mice. The mice were divided into two groups: no exercise (non-exercise group) and 8-weeks of aerobic exercise training (exercise group), with six mice in each group. Dual-energy X-ray absorptiometry and micro-computed tomography showed that femoral and tibial bone parameters improved after aerobic exercise training. Greater skeletal nerve fiber density was also observed in the distal femoral and proximal tibial periostea, measured and analyzed by immunofluorescence staining and confocal microscopy. Pearson correlation analysis revealed a significant association between skeletal nerve densities and trabecular bone volume/total volume ratios (distal femur; *R*^2^ = 0.82, *p* < 0.05, proximal tibia; *R*^2^ = 0.59, *p* = 0.07) in the exercise group; while in the non-exercise group no significant correlation was found (distal femur; *R*^2^ = 0.10, *p* = 0.54, proximal tibia; *R*^2^ = 0.12, *p* = 0.51). Analysis of archival microarray database confirmed that aerobic exercise training changed the microRNA profiles in the mice femora. The differentially expressed microRNAs reinforce the role of aerobic exercise training in the osteogenic and neurogenic potential of femora and tibiae. In conclusion, 8-weeks of aerobic exercise training positively regulate bone metabolism, an effect that paralleled a significant increase in skeletal nerve fiber density. These findings suggest that aerobic exercise training may have dual utility, both as a direct stimulator of bone remodeling and a positive regulator of skeletal nerve regeneration.

## Introduction

Advancing age is a well-known irreversible risk factor for bone loss and increased fracture risk associated with osteoporosis (Aspray and Hill, 2019). Osteoporosis may be considered an imbalance in bone remodeling in which the degree of bone-formation (osteoblast activity) is less than that of bone resorption (osteoclast activity) (Eastell et al., 1993). Osteoporosis can cause chronic pain, diminish the quality of life (Lips and van Schoor, 2005), increase the healthcare burden (Singer et al., 2015), and mortality (Cauley et al., 2000; Bliuc et al., 2009) of patients. Pharmacological treatment options for osteoporosis and fracture prevention in the elderly are available, however, non-pharmacological approaches to prevent age-associated bone loss and osteoporosis are required.

Exercise is a renowned non-pharmacological intervention that effectively improves bone metabolism by stimulating bone adaptation to mechanical force (Santos et al., 2017). Exercise-induced mechanical force stimulates mechano-sensors and osteocytes, which control bone remodeling by regulating osteoblast and osteoclast differentiation (Klein-Nulend and Bakker, 2007) and stimulating osteoprotegerin, which inhibits osteoclastogenesis (Regard et al., 2012). Exercise also has several indirect effects, such as enhancing bone vascular function and blood flow. These alter the osteoblast and osteoclast differentiation *via* the nitric oxide (NO)-mediated pathway (Dominguez et al., 2010; Stabley et al., 2014). Several studies have reported the beneficial effects of exercise on bone mineral density (BMD) and other bone properties in mature animals (Isaksson et al., 2009; Thongchote et al., 2014; Kanazawa et al., 2020). In addition, emerging evidence suggests that exercise plays a crucial role in minimizing the effects of inflammation (Kasapis and Thompson, 2005; Pastva et al., 2005) and promoting peripheral nerve regeneration (Sabatier et al., 2008; Park and Höke, 2014). Exercise training has also been shown to protect against peripheral neuropathy in diabetic (Kluding et al., 2012) and chemotherapy-induced (Park et al., 2015) peripheral neuropathy models.

Recently, increasing evidence has suggested that peripheral afferent nerve fibers in bone tissue regulate the osteoanabolic activity in load-induced bone formation (Tomlinson et al., 2017). The mechanical loading-induced anabolic response requires peripheral afferent neurons that express tropomyosin receptor kinase A (TrkA) in the skeleton (Tomlinson et al., 2017). Furthermore, nerve growth factor (NGF) expression and concurrent early invasion of TrkA-expressing skeletal nerves regulate bone anabolic activity during the repair process (Li Z. et al., 2019; Meyers et al., 2020) and stimulate trauma-induced heterotopic ossification progression (Lee et al., 2021). Previous studies have demonstrated the positive effects of exercise on BMD, bone strength (Johansson et al., 2015; Abe et al., 2016) and peripheral nerves, such as peripheral neuropathy prevention and peripheral nerve regeneration (Sabatier et al., 2008; Park and Höke, 2014; Park et al., 2015). However, mechanistic details regarding the association between skeletal nerves, bone mineralization, trabecular bone microarchitecture, and aerobic exercise training are lacking. In this brief research report, we investigated the association between skeletal nerve fibers and changes in trabecular and cortical bone of the femur and tibia in middle-aged mice following an 8-week aerobic exercise training regimen, and how this relationship is linked to age-associated bone changes.

## Materials and Methods

### Animals

Since bone structure and function is generally preserved in male mice up to 18 months of age (Aslam et al., 2016), middle-aged mice were considered appropriate for determining the relationship between trabecular bone microarchitecture and skeletal nerves following aerobic exercise training. Twelve male C57BL/6 mice were purchased from Daehan Biolink (Daejeon, South Korea) and were housed in standard cages at a pathogen-free animal care facility at a temperature of 22 ± 2°C and humidity of 50%, in a 12:12-h light-dark controlled room. Tap water and standard chow (Purina Mills, Seoul, South Korea) were provided *ad libitum*. All mice were weighed weekly.

### Ethics Statement

The animal experimental protocol was approved by the Institutional Animal Care and Use Committee (IACUC) of the Sungkyunkwan University School of Medicine (SKKUIACUC 18-5-24-3). We followed the AAALAC International Guidelines for Animal Experiments.

### Study Design and Exercise Protocol

Following a 1-week acclimation period, all mice (aged 14 months) were randomly assigned to either the non-exercise (*n* = 6) or aerobic exercise (*n* = 6) groups. The mice in the aerobic exercise group performed treadmill running on a motor-driven treadmill (Columbus Instruments, Inc., Columbus, OH, United States). Five days prior to the initiation of the study, mice in the aerobic exercise training group were acclimated to treadmill running for up to 30 min at 5–10 m/min. For the entire 8-week experimental period, the aerobic exercise group ran on the treadmill for 30 min at a fixed speed of 18 m/min 5 days/week. Mice in the non-exercise group were not exposed to treadmill exercise, and instead performed routine daily activities in housing cages. The intensity of exercise performed was determined based on a percentage of maximal oxygen consumption (%VO2max) index published previously (Wasityastuti et al., 2019). Exercise was performed at an intensity of 85% VO_2max_. The 8-week period of exercise training is considered long-term training, and is approximately equivalent to 6 human years, based on a lifespan comparison between mice and humans (Dutta and Sengupta, 2016). Each treadmill session included 5-min (at 5 m/min) warm-up and cool-down periods. Mice were encouraged to run with a gentle tap on the tail, and no electrical shock was administered.

After completion of the exercise protocol, all mice were anesthetized *via* an intraperitoneal injection of a mixture of Zoletil (40 mg/kg) and Rompun (5 mg/kg) (Cho et al., 2015). Mice were placed in the anesthesia chamber until they lost their reflex after a tail pinch test. Following anesthesia, all mice were euthanized by cardiectomy. The right femora and tibiae were harvested and placed in 4% paraformaldehyde (PFA) at 4°C for 24 h. These were subsequently scanned using dual-energy X-ray absorptiometry (DXA) and micro-computed tomography (μCT).

### DXA Scan

Dual-energy X-ray absorptiometry scans and analyses were performed using UltraFocus DXA equipment (UltraFocus DXA, Faxitron Bioptics, LLC., Tucson, AZ, United States) and small animal-specific software. The whole femora and tibiae were scanned and analyzed to measure the total bone area, BMD, and bone mineral content (BMC).

### μCT Imaging and Analyses

**FIGURE 1:**
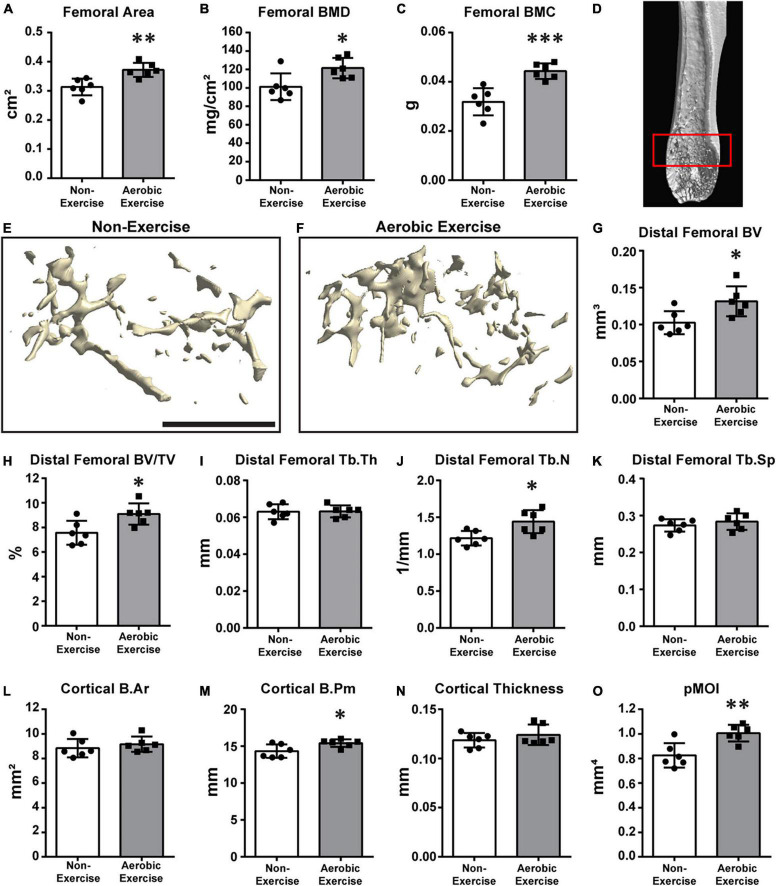
Higher distal femoral BMD, BMC, trabecular bone microarchitecture, and cortical bone perimeter following aerobic exercise training. Quantification of area, BMD, BMC of distal femora **(A–C)**. Femoral area was higher (*p* < 0.01) in Aerobic Exercise group vs. Non-Exercise group **(A).** Femoral BMD (*p* < 0.05) and BMC (*p* < 0.001) were significantly greater in mice with aerobic exercise compared to mice without exercise training **(B,C)**. Representative 3D μCT reconstruction images of region of interest [**(D)** red box] and distal femoral trabecular bone microarchitecture in Non-Exercise and Aerobic Exercise groups **(E,F).** Distal femoral BV was higher (*p* < 0.05) in Aerobic Exercise group vs. Non-Exercise group **(G)**. Femoral BV/TV was significantly greater in mice with aerobic exercise compared to Non-Exercise group (*p* < 0.05) **(H).** Femoral Tb.N was significantly higher (*p* < 0.05) in Aerobic Exercise group compared to Non-Exercise group **(J)**. Cortical B.Pm **(M)** and pMOI **(O)** of distal femur were significantly higher with aerobic exercise, while trabecular Tb.Th **(I)**, Tb.Sp **(K)**, Cortical B.Ar **(L)**, and Cortical thickness **(N)** did not differ between groups. Black scale bar: 1 mm. *N* = 6 per group. Values are means ± S.D. *p < 0.05, ^**^p < 0.01, and ^***^p < 0.001.

**FIGURE 2:**
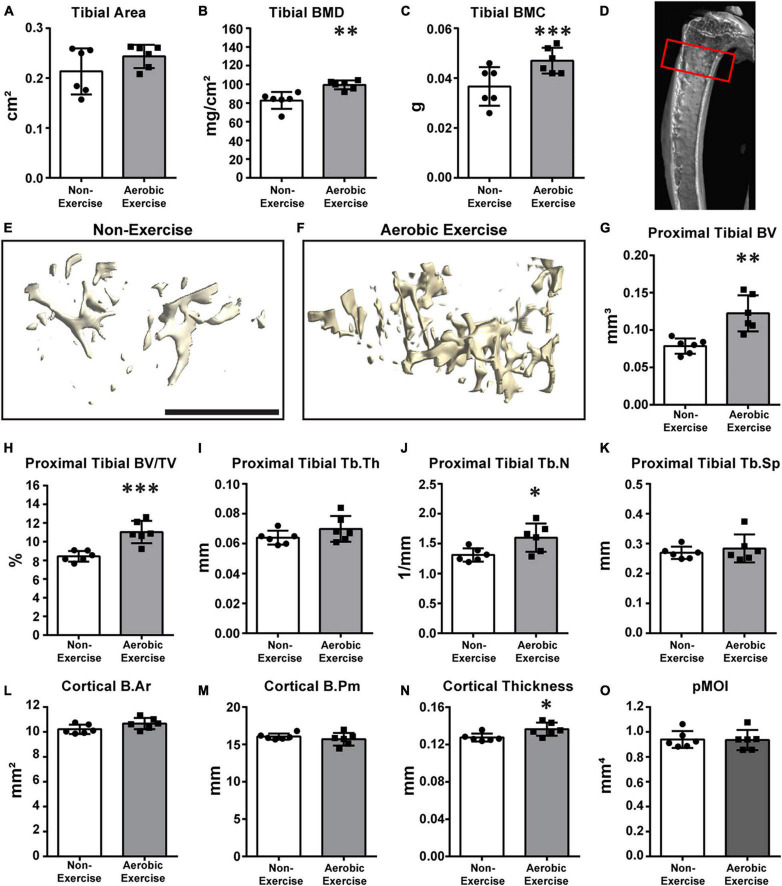
Aerobic exercise training demonstrates greater proximal tibial BMD, BMC, trabecular bone microarchitecture, and cortical bone perimeter. Quantification of area, BMD, and BMC of proximal tibiae **(A–C)**. Tibial area did not differ between groups **(A)**. Tibial BMD [**(B)** *p* < 0.01] and BMC [**(C)** *p* < 0.001] of mice in Aerobic Exercise group were significantly different from mice in the Non-Exercise group. Representative 3D μCT reconstruction images of region of interest [**(D)** red box] and proximal tibial trabecular bone microarchitecture in Non-Exercise and Aerobic Exercise groups **(E,F)**. Proximal tibial BV was significantly higher (*p* < 0.01) in Aerobic Exercise group vs. Non-Exercise group **(G)**. Tibial BV/TV was also greater (*p* < 0.001) with aerobic exercise training compared to no exercise **(H)**. Tibial Tb.Th **(I)** and Tb.Sp **(K)** were not different between groups. Tibial Tb.N was higher with aerobic exercise vs. without exercise **(J)**. Cortical thickness was significantly thicker in aerobic exercise group vs. non-exercise group **(N)**. Cortical B.Ar tended to be higher with aerobic exercise [**(L)** *p* = 0.08], while Cortical B.Pm **(M)** and pMOI **(O)** were not different. Black scale bar: 1 mm. *N* = 6 per group. Values are means ± S.D. *p < 0.05, ^**^p < 0.01, and ^***^p < 0.001.

The femora and tibiae were dissected, skin and muscle were removed, and bone was evaluated using a SkyScan1172 high-resolution μCT imaging system (Bruker, Kontich, Belgium). Scans were obtained at an image resolution of 10 μm, using the following settings: 1 mm aluminum filter, 65 kVP X-ray voltage, 153 μA anode current, 65 ms exposure time, 5-frame averaging, and 0.3° rotation step. Metaphyseal trabecular bone microarchitecture of the distal femora and proximal tibiae were determined as previously described (Lee and Prisby, 2018). Briefly, 60 slices were made in the distal femoral and 50 slices in the proximal tibial metaphyses, beginning at 600 μm superior and 500 μm inferior to the growth plate, respectively (Figures 1D, 2D, respectively). The following trabecular bone microarchitectural parameters were calculated: bone volume (BV, mm^3^), bone volume/total volume ratio (BV/TV,%), trabecular thickness (Tb.Th, μm), trabecular number (Tb.N,/mm^2^), and trabecular separation (Tb.Sp, μm). Cortical bone parameters including cortical bone area (B.Ar, mm^2^), cortical bone perimeter (B.Pm, mm), cortical thickness (mm), and polar moment of inertia (pMOI, mm^4^) were also determined in the same region as the trabecular bone. Care was taken not to include the trabecular bone in the cortical bone analysis. Reconstructed three-dimensional (3D) images were generated *via* the Feldkamp algorithm using a commercially available NRecon software package (2.0.4.0 SkyScan, Kontich, Belgium), and 3D morphometric imaging and analyses were performed using the CTAn and CTVox (1.13 SkyScan, Kontich, Belgium) software.

### Histology and Immunohistochemistry

After radiological analyses were performed, bones were processed for histology and immunohistochemistry as described in previous studies (Li Z. et al., 2019; Meyers et al., 2020; Lee et al., 2021). The femora and tibiae were decalcified in 14% ethylenediaminetetraacetic acid (EDTA, Sigma-Aldrich, St. Louis, MO, United States) for 4 weeks, the samples were cryoprotected in 30% sucrose for 24 h at 4°C and then embedded in optimal cutting temperature compounds (O.C.T; Tissue-Tek 4583, Torrance, CA, United States). Frontal sections of tissues were obtained *via* cryostat sectioning at either 10 or 50 μm thickness and mounted on slides (Fisherbrand™ Superfrost Plus™, Fisher Scientific, Waltham, MA, United States). For immunofluorescence staining, sections were washed for 15 min in phosphate buffered saline (PBS) three times. Next, the samples were permeabilized with 0.5% Triton-X for 30 min and blocked with 5% normal goat serum for 30 min. Tissue sections were then incubated with anti-beta tubulin 3 (Anti-TUBB3, Abcam Ab18207, 1:1,000 volume) overnight at 4°C. After overnight incubation, samples were washed in PBS three times for 15 min and incubated with the secondary antibody (A594-Goat Anti-Rabbit IgG) for 1 h at room temperature. Samples were mounted with 4′,6-diamidino-2-phenylindole (DAPI) mounting solution (Vectashield H-1800, Vector Laboratories, Burlingame, CA, United States). Fluorescent images were obtained using a confocal microscope (Zeiss LSM780 FCS, Carl Zeiss Microscopy GmbH, Jena, Germany).

### Histology Image Analysis

Beta III tubulin (TUBB3)^+^ nerve fibers were quantified using Imaris software v9.3 (Oxford Instruments, Belfast, United Kingdom) using a previously described 3D volumetric analysis method (Li Z. et al., 2019; Meyers et al., 2020; Lee et al., 2021). Within the periosteum of the distal femoral and proximal tibial metaphyses, we analyzed seven longitudinal serial fields per sample. A consistent region centered on the boundary between the metaphysis and growth plate was used, including the metaphyseal periosteum and cortical bone.

### Microarray

Microarray data from 4-month-old male BALB/c mice femora with- and without 8 weeks of treadmill running were obtained from publicly available Gene Expression Omnibus (GEO) dataset GSE179201. In this study, randomly allocated male BALB/c mice (*n* = 3/group) underwent either 8 weeks or no treadmill running. After the exercise training session, total RNAs were extracted from femora. The expression profile of a total of 1,900 microRNAs (miRNAs) was attained by using miRNA microarray. All bioinformatics analyses and normalization were performed with GraphPad Prism v9 software with subsequent principal component analysis visualization. All data underwent log2 transformation, and *p*-values were adjusted for the false discovery rate using Benjamini-Hochberg procedure.

### Statistics

Parametric data were analyzed using unpaired Student’s *t*-test to examine differences between groups. Sample sizes of experiments presented in Figures 1, 2 (BMD and BV/TV, %) were calculated using G*Power v 3.1.9.2 software based on an anticipated effect size (F) of 0.72, which was determined based on previously published mouse hindlimb bone and exercise data (Takagi et al., 2017). In a scenario in which there are six replicates per group, the difference between two independent means *via* Student’s *t*-test would provide 80% power to detect effect sizes of 1.60, assuming a two-sided significance level of 0.05. Bivariate associations between periosteal nerve fiber density and the trabecular BV/TV of the distal femur and proximal tibia were analyzed using Pearson correlation coefficients. All statistical analyses were performed using GraphPad Prism v6.01 and v9 software (GraphPad Software, Inc., San Diego, CA, United States). Quantitative data are expressed as mean ± SD, with values of *p* < 0.05 considered significant.

## Results

### Animal Characteristics Following Aerobic Exercise Training

Pre- and post-exercise body, heart, muscle, and epididymal fat mass were examined (Supplementary Table 1). No differences in mean body mass were observed at baseline (non-exercise group: 34.6 ± 1.7, aerobic exercise group: 35.0 ± 1.9) or post-exercise (non-exercise group: 36.0 ± 1.9, aerobic exercise group: 34.2 ± 2.4) between the groups. Although the differences were not statistically significant, body mass was slightly lower after exercise training compared to baseline in the exercise group, while the mean body mass of the non-exercise group increased slightly. Heart and muscle masses were slightly higher in the aerobic exercise group than in the non-exercise group, although the differences were not statistically significant. Epididymal fat mass was significantly lower after aerobic exercise training (non-exercise group: 1,117.5 ± 219.2 and aerobic exercise group: 734.9 ± 293.8, *p* < 0.05).

### Distal Femoral Bone Mineral Density, Bone Mineral Content, Trabecular Bone Microarchitecture, and Cortical Bone

We determined the area, BMD, BMC, trabecular bone microarchitecture, and cortical bone parameters of the distal femur (Figure 1). Then, the values determined for middle-aged mice subjected to aerobic exercise for 8 weeks were compared with those from mice that did not perform exercise. In mice of the exercise group, femoral area was 19% larger than that of the non-exercise group (Figure 1A, *p* < 0.01). Similarly, the BMD and BMC of the exercise group were 20 and 39% higher than those of the non-exercise group, respectively (Figure 1B, *p* < 0.05; and Figure 1C, *p* < 0.001). Figure 1D shows the representative image of the region of interest (ROI). We also determined the effect of aerobic exercise on the trabecular bone microarchitecture of the distal femora using μCT 3D reconstruction (Figures 1E,F). Distal femoral trabecular BV and BV/TV values of the exercise group were 28 and 20% higher than those of the non-exercise group, respectively (Figures 1G,H, *p* < 0.05). The increased trabecular BV was mainly due to an 18% increase in the trabecular number of the distal femur in the exercise group (Figure 1J, *p* < 0.05). However, trabecular thickness and separation were not different (Figures 1I,K). Cortical B.Ar and cortical thickness were not different between the groups (Figures 1L,N). Cortical B.Pm demonstrated a mean 8% increase in the aerobic exercise group in comparison to the non-exercise group (Figure 1M, *p* < 0.05). This resulted in a 22% increase in the bone strength parameter pMOI with aerobic exercise (Figure 1O, *p* < 0.01).

### Proximal Tibial Bone Mineral Density, Bone Mineral Content, Trabecular Bone Microarchitecture, and Cortical Bone

We then determined whether aerobic exercise training increased BMD, BMC, trabecular bone microarchitecture, or cortical bone in the proximal tibia (Figure 2). Although the tibial area did not differ (Figure 2A), tibial BMD and BMC values of the aerobic exercise group were 20 and 35% higher than those of the non-exercise group, respectively (Figures 2B,C; *p* < 0.001 for both). Figure 2D shows the representative image of the ROI. Similar to distal femoral trabecular bone microarchitecture findings, meaningful differences in proximal tibial trabecular bone microarchitecture were observed when values of the aerobic exercise and non-exercise groups were compared. μCT 3D reconstructions of trabecular bone microarchitecture in the secondary spongiosa of the proximal tibial metaphyses revealed that the trabecular bone parameters, BV, BV/TV, and Tb.N, of the aerobic exercise group were higher than those of the non-exercise group (Figures 2E,F) by 56, 31, and 22%, respectively (Figure 2G, *p* < 0.001; Figure 2H, *p* < 0.001; and Figure 2J, *p* < 0.05). Similar to the distal femur, trabecular thickness and separation were not different (Figures 2I,K). Interestingly, in the proximal tibia, cortical bone changes differently when compared to the distal femur. While the B.Pm and pMOI of the cortical bone did not change (Figures 2M,O), cortical B.Ar and cortical thickness were increased by 4.5 and 7%, respectively (Figure 2L, *p* = 0.08 and Figure 2N, *p* < 0.05, respectively).

### Periosteal Nerve Fiber Density Increased After 8 Weeks of Aerobic Exercise and Strongly Associated With Trabecular Bone Volume

**FIGURE 3:**
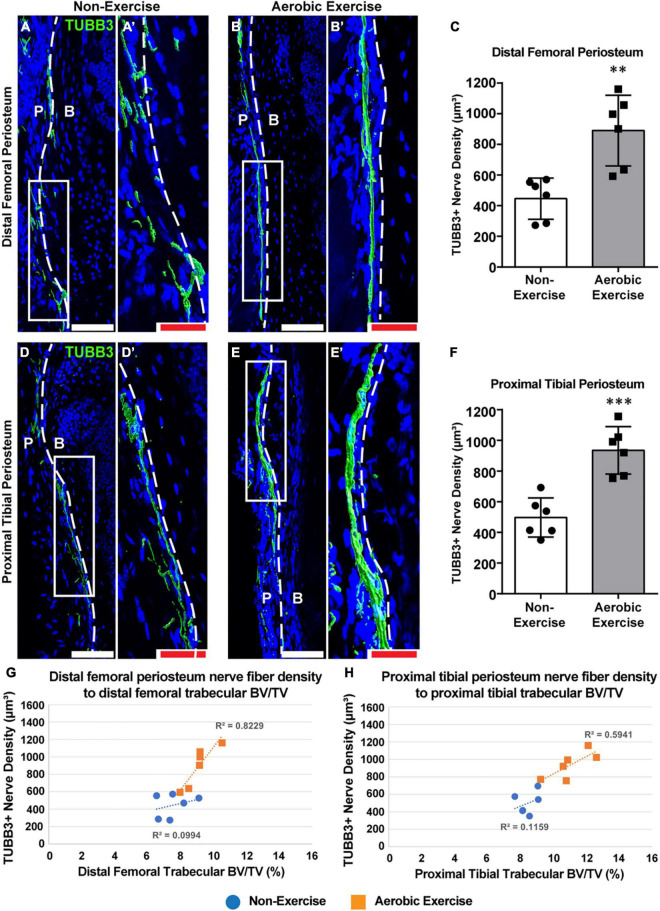
Aerobic exercise training shows greater distal femoral and proximal tibial periosteal innervation with strong association with trabecular bone volume. Representative images of pan-neuronal beta III tubulin (TUBB3) immunohistochemical staining in distal femoral periosteum of Non-Exercise **(A)** and Aerobic Exercise **(B)**. Higher magnification images were shown **(A′,B′)**. Quantification of TUBB3 immunohistochemical staining in the distal femoral periosteum showed higher (*p* < 0.01) TUBB3^+^ nerve fiber density in Aerobic Exercise group **(C)**. Representative images of pan-neuronal beta III tubulin (TUBB3) immunohistochemical staining in proximal tibial periosteum of Non-Exercise **(D)** and Aerobic Exercise **(E)**. Higher magnification images were shown **(D′,E′)**. Quantification of TUBB3 immunohistochemical staining in the proximal tibial periosteum revealed greater (*p* < 0.001) TUBB3^+^ nerve fibers following aerobic exercise **(F)**. TUBB3 immunohistochemical staining appears green using the surface rendering function of Imaris analyzing software. Scattergrams showing the association between TUBB3^+^ periosteal nerve fiber density and trabecular BV/TV in the distal femora **(G)** and in the proximal tibiae **(H)** with Non-Exercise group (blue) or Aerobic Exercise group (orange). Statistically significant and strong correlation exists (*R*^2^ = 0.82, *p* = 0.012) between periosteal nerve fiber density and trabecular BV/TV in femora [**(G)** orange], while not statistically significant but strong relationship (*R*^2^ = 0.59, *p* = 0.072) was observed between nerve fiber density and trabecular BV/TV in tibiae [**(H)** orange] with aerobic exercise training. No correlations exist between skeletal nerve fibers and femoral and tibial trabecular BV/TV in Non-Exercise groups [**(G,H)** blue]. Dashed white line represents a boundary between the periosteum (P) and adjacent cortical bone (B). White scale bar: 100 μm, red scale bar: 50 μm. *N* = 6 per group. Values are means ± S.D. ^**^p < 0.01, and ^***^p < 0.001.

We determined the effect of the 8-week aerobic exercise protocol on skeletal nerve fibers. Nerve fiber densities of the distal femoral and proximal tibial periostea were analyzed in experimental mice (Figure 3). For both the distal femoral and proximal tibial periostea, nerve fiber density was greater in mice of the exercise group than in the non-exercise group (Figures 3A,B,D,E). The density of TUBB3^+^ nerve fibers within periostea was quantified, which revealed that for mice of the exercise, 99 and 88% increase in nerve fiber density in the distal femoral and proximal tibial periosteum was observed, respectively (Figure 3C, *p* < 0.01; Figure 3F, *p* < 0.001). Thus, the aerobic exercise training program increased skeletal nerve density in the periosteum of the distal femur and proximal tibia. Associations between skeletal nerve fibers and trabecular bone volume in the secondary spongiosa of the distal femora and proximal tibiae were explored *via* bivariate analysis. Significant correlations were observed between periosteal nerve fiber density and trabecular bone volume (*R*^2^ = 0.82, *p* = 0.012) in the distal femora in mice of the exercise group were considered, while no correlation was observed in the non-exercise group (*R*^2^ = 0.09, *p* = 0.54) (Figure 3G). Although not statistically significant, a correlation was observed between skeletal nerve fiber density and trabecular bone volume (*R*^2^ = 0.59, *p* = 0.072) in the proximal tibiae for the aerobic exercise training group, while variables were more weakly associated in the non-exercise group (*R*^2^ = 0.11, *p* = 0.51) (Figure 3H).

### Differentially Expressed miRNA Following Aerobic Exercise in Femora

**FIGURE 4:**
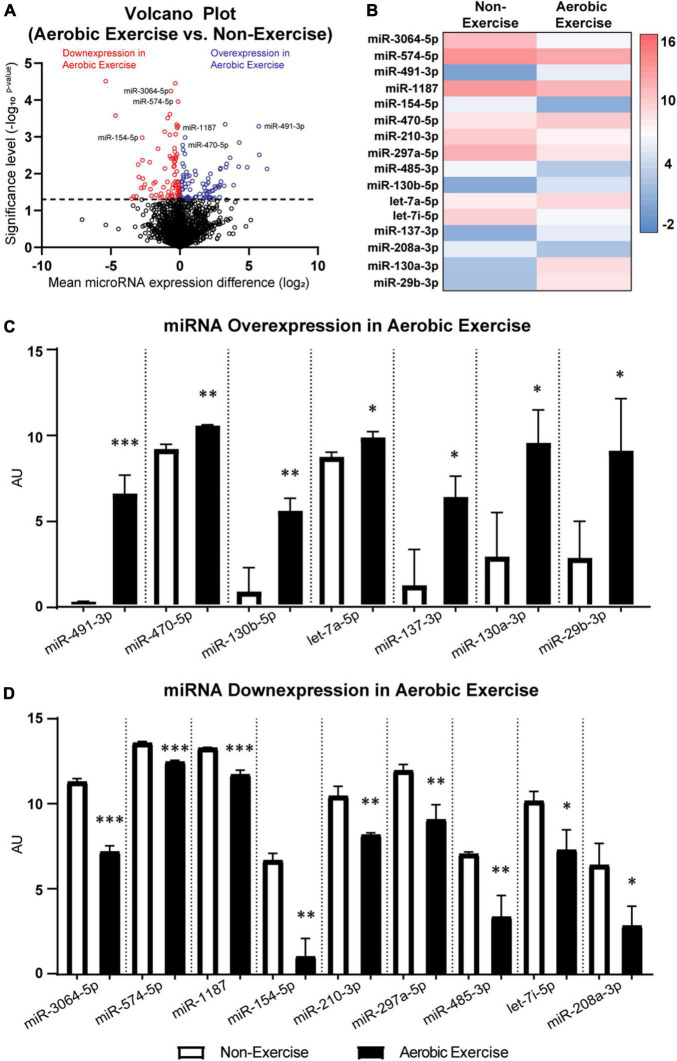
MicroRNA differentially expressed in femora of 4-month-old male BALB/c mice following 8 weeks of aerobic exercise training. Volcano plot of differentially expressed miRNAs with- and without aerobic exercise training **(A)**. 191 out of 1,900 miRNAs showed statistically significant expression differences between non-exercise and aerobic exercise (above the dotted line). Altogether 91 out of 191 miRNAs had increased expression (blue color), while 100 miRNAs showed decreased expression following aerobic exercise (red color). Particularly, miRNAs that are associated with bone and nerve were differentially expressed in femora following aerobic exercise **(B)**. Over-expressed miRNAs **(C)** and down-expressed miRNAs **(D)** following aerobic exercise training are depicted. See Supplementary Tables 2, 3 for the predictive actions of miRNAs. *N* = 3 per group. Values are means ± S.D. *p < 0.05, ^**^p < 0.01, and ^***^p < 0.001.

The potential upstream mediators of osteogenesis and neurogenesis in mice femora were assessed. Previous literature explored the effects of 8-week treadmill exercise training (13 m/min; 9° slope; for 40 min/day; 6 days/week) on the miRNA expression in the femora of 4-month-old male BALB/c mice (Yang et al., 2021). Re-examination of the microarray dataset (GSE179201) shown in Figure 4A revealed that a total of 191 out of 1,900 miRNAs were differentially expressed (*p* < 0.05) between the non-exercise and aerobic exercise training groups. Ninety-one miRNAs were over-expressed, while 100 miRNAs were down-expressed with aerobic exercise training when data were normalized by non-exercise control group. Particularly, 16 miRNAs that are related to osteogenic and neurogenic regulation were selected among the differentially expressed miRNAs (Figure 4B). Expression levels of seven associated upstream osteogenesis and neurogenesis non-coding RNAs including miR-491-3p, miR-470-5p, miR-130b-5p, let-7a-5p, miR-137-3p, miR-130a-3p, and miR-29b-3p were upregulated in the aerobic exercise group compared to the non-exercise group (Figure 4C). The other osteogenic and neurogenic miRNAs including miR-3064-5p, miR-574-5p, miR-1187, miR-154-5p, miR-210-3p, miR-297a-5p, miR-485-3p, let-7i-5p, and miR-208a-3p were downregulated with the aerobic exercise (Figure 4D). The potential actions and contributions of the 16 miRNAs in bone metabolism and nervous system were predicted, and the detailed predictive actions of miRNAs are presented in the Supplementary Tables 2, 3.

## Discussion

Osteoporosis is a devastating age-related metabolic disease associated with low BMD, BMC, reduced bone volume and bone microstructure caused by dysfunctional/dysregulated bone remodeling, leading to increased fracture risk (Aspray and Hill, 2019). Exercise training is one of the options of alternative non-pharmacological treatment. Despite this known factor, evidence for the underlying mechanisms of the prevention of age-associated bone change due to aerobic exercise is equivocal. There has been growing attention to the diverse roles that skeletal nerves play in bone health. Previous studies in animal models have shown that skeletal nerves play a significant role in bone development (Tomlinson et al., 2016), bone fracture or repair (Li Z. et al., 2019; Meyers et al., 2020), neuropathic disease-related bone loss (Li Z. et al., 2019), and ectopic bone formation after traumatic injury (Lee et al., 2021). Furthermore, the role of exercise in the prevention of peripheral neuropathy (Kluding et al., 2012; Park et al., 2015) and improved peripheral nerve regeneration (Sabatier et al., 2008; Park and Höke, 2014) has been documented. However, the association between skeletal nerves and bone metabolism, particularly during aerobic exercise training, has not been evaluated. In this study, we showed that skeletal nerve fiber density, BMD, BMC, trabecular bone microarchitecture, and cortical bone parameters were higher in the group that underwent aerobic exercise training than in the group that did not. To our knowledge, this is the first study to report that aerobic exercise training increases skeletal nerve fiber density both in the femora and tibiae. We firmly believe that this novel finding provides a good starting point from which further research may examine whether exercise-skeletal nerve interactions improve bone remodeling during the process of aging.

Findings from the current study are consistent with prior findings that explored aerobic exercise training-induced peripheral nerve regeneration in mice (Park and Höke, 2014). Park and Hoke discovered that both axonal number and size within the distal median nerve in the upper forelimb of experimental animals were augmented compared to non-exercise control mice (Park and Höke, 2014). In the present study, hindlimb BMD, BMC, trabecular bone microarchitecture, and cortical bone in the distal femur and proximal tibia were greater in aerobic exercise-trained than in the control mice. The protective role of exercise training against chronic diseases such as osteoporosis, diabetes, and cardiovascular diseases and the subsequent improvement in health status have been documented (Warburton et al., 2006). Findings of the current study are in accordance with those that have been reported previously, and suggest that 8 weeks of aerobic exercise training improves BMD, BMC, trabecular bone volume, and cortical bone parameters in middle-aged mice. The mechanism by which aerobic exercise can stimulate an increase in trabecular bone metabolism and cortical bone size may be related to prolonged periods of intense mechanical force during the treadmill exercise, which facilitates dynamic changes of bone interstitial fluid flow (Cowin and Cardoso, 2015). Indeed, changes in flow of interstitial fluid by mechanical loading further promote osteoblasts and osteoclasts activation and stimulate osteoprotegerin (Klein-Nulend and Bakker, 2007; Regard et al., 2012; Santos et al., 2017). However, the relative importance of skeletal nerves in bone remodeling remains unclear and we cannot rule out the possibilities that improved trabecular and cortical bone parameters could have been achieved *via* increased innervation.

The spatial distribution, innervation, and functional role of the skeletal sensory nerves have been strongly implicated in bone development and healing. The most common nerve fibers in the skeleton are TrkA-expressing sensory nerve fibers (Jimenez-Andrade et al., 2010; Castañeda-Corral et al., 2011). Sensory nerves have previously been linked to long bone development during embryogenic and prenatal phases (Tomlinson et al., 2016), ulnar stress fracture healing (Li Z. et al., 2019), calvaria bone defect repair (Meyers et al., 2020), and the abnormal formation of ectopic bone after traumatic injury (Lee et al., 2021). In contrast, a previous study demonstrated that sympathetic neuronal projections increase levels of receptor activator of nuclear factor kappa-B ligand (RANKL) and stimulate osteoclast activity, which regulates bone resorption and bone catabolism (Chen et al., 2019). The strong association between periosteal nerve fiber density and trabecular bone volume with aerobic exercise training in this study suggests a physiological link between nerve signaling and bone remodeling following exercise training. Therefore, one may speculate that neuronal mechanisms influence skeletal outcomes and that aerobic exercise training may function as a nerve stimulant.

The molecular mechanisms by which nerves of the periosteum regulate bone cell activity and function have not been fully elucidated. Nor have mechanistic details surrounding the aerobic exercise-mediated induction of nerve-bone interactions. Prolonged periods of intense mechanical force during treadmill exercise likely increase the levels of transmission of neurotrophic factors and neuropeptides, such as NGF and neuregulin (NRG)-1. Based on the load-induced bone formation model, axial compression of the forelimb upregulates the expression of NGF in osteoblasts on the surface of the ulnar periosteum, which guides tropomyosin receptor kinase A (TrkA)^+^ nerve fiber sprouting (Tomlinson et al., 2017). Loss of NGF and TrkA signaling pathways also reduces load-induced nerve invasion and impairs Wnt/β-catenin signaling, which may reduce levels of bone formation (Tomlinson et al., 2017). Furthermore, the significance of the NRG1 and receptor Erb in cellular activity of the bone has been documented. Moderate-intensity treadmill exercise training activates NRG1/ErbB signaling (Cai et al., 2016), and Wnt3a-mediated upregulation of Erb promotes osteoblast differentiation and proliferation (Jullien et al., 2012). Therefore, the long-term mechanical force generated by 8 weeks of aerobic exercise may accelerate the NGF-TrkA and NRG1-Erb signaling pathways and stimulate bone formation *via* the Wnt/β-catenin signaling pathway. Nevertheless, the identification of neuronal factors that play a principal role in aerobic exercise-associated bone formation is needed.

The various differentially expressed miRNAs selected in the current study demonstrate the potential role of exercise in the positive regulation of osteogenesis and neurogenesis in the skeleton. This appears to involve the miRNA-130b-5p, over-expression of which induces neural progenitor cell proliferation and attenuates neuronal cell apoptosis in the central nervous system (brain and spinal cord) (Jönsson et al., 2015; Zhang et al., 2018). Similarly, miR-130a-3p over-expression promotes the Vascular Endothelial Growth Factor Receptor-2 expression in the sensory dorsal root ganglia (DRG) and regulates the peripheral nerve system by stimulating axonal growth (Glaesel et al., 2020). The overexpression of miR-130a-3p also regulates bone metabolism, which in turn increases osteogenic differentiation of adipose-derived stem cells by enhancing the Wnt//β-catenin signaling pathway (Yang et al., 2020). On the other hand, down-expression of numerous miRNAs following aerobic exercise positively effects ossification and neurogenesis. For example, miR-3064-5p inhibits osteoblastic differentiation and reduces the expression of osteogenic biomarkers, including osteocalcin (OCN), osteopontin (OPN), Runx2, and alkaline phosphatase (ALP) (Huang et al., 2021). In an experimental model, knock-down of miR-3064-5p promoted osteoblast differentiation and osteoblast-associated gene expression (Huang et al., 2021), which suggests that down-expression of miR-3064-5p following aerobic exercise may upregulate the osteoblast-associated bone formation. Likewise, increased miR-485-3p suppresses neural stem cells (NSC) growth, proliferation, and differentiation, and increases neuroinflammation; therefore, knock-down of miR-485-3p expression promotes neuronal viability and neuro-regeneration, and reduces neuroinflammation (Gu et al., 2020; Yu et al., 2021). In particular, down-expression of certain miRNAs following exercise training may play a crucial role in osteogenic and neurogenic potential in our exercise model. Nonetheless, the exact physiologically important downstream gene and molecular mechanisms which stimulate osteogenesis and neurogenesis in the bone remain unknown, and are subjects of further study.

Some caveats exist in the extrapolation of our results. First, the omission of baseline data, other than body mass, limits our ability to truly describe whether aerobic exercise training improved BMD, BMC, trabecular bone microarchitecture, and cortical bone in the distal femur and proximal tibia. However, comparisons between mice of the non-exercise and aerobic exercise groups, which were of the same species, strain, and age, minimized confounding variables and enabled us to conclude that increased BMD, BMC, trabecular and cortical bone parameters were due to aerobic exercise training. However, since this is the first study revealing a connection between aerobic exercise training, bone formation, and skeletal innervation in middle-aged mice, we believe that these studies are timely and highly impactful. Thus, mechanical force-derived bone cellular activity, recruitment, and new bone formation, including bone histomorphometry with Goldner’s trichrome, staining of tartrate-resistant acid phosphatase (TRAP), and dynamic properties following aerobic exercise training should be assessed in future studies. The sample size in this study was relatively small, which limits our ability to neglect effects of animal-to-animal variation. However, mice of the same species, strain, and age were randomly assigned to the two groups, and all mice were housed in similar environments, which strengthened research animal randomization and minimized the potential for animal-to-animal variation. Importantly, statistically significant differences in bone and nerve parameters were observed despite the small sample size.

Our results demonstrate that mice exposed to aerobic exercise training for 8 weeks had greater BMD, BMC, trabecular bone microarchitecture, and cortical bone values in the distal femur and proximal tibia than those that did not perform exercise. These changes were accompanied by increases in skeletal nerve fiber density in the long bones of mice. These results suggest that skeletal nerve density increase following aerobic exercise training is linked to an increase in trabecular volume and cortical bone in long bones in middle-aged mice. Our aggregate data suggest that aerobic exercise training may have dual utility in the elderly, both as a direct stimulator of bone remodeling and a positive regulator of skeletal nerve stimulation. The extent to which aerobic exercise training-related increase in skeletal innervation contributes to improvement in bone metabolism remains an intriguing and unanswered question.

## Data Availability Statement

Microarray data from 4-month-old male BALB/c mice femora with and without 8 weeks of treadmill running were obtained from publicly available Gene Expression Omnibus (GEO) dataset “GSE179201” at https://www.ncbi.nlm.nih.gov/geo/. All other relevant data and the original contributions presented in the study are included in the article/Supplementary Material, and any further inquiries can be directed to the corresponding authors.

## Ethics Statement

The animal study was reviewed and approved by Sungkyunkwan University School of Medicine (SKKUIACUC 18-5-24-3).

## Author Contributions

SL, Y-AS, JC, D-HP, and CK contributed to the conception and design of the study, and the data interpretation, commented on the previous versions of the manuscript, and read and approved the final manuscript. SL and JC performed the material preparation, data collection, and analysis. SL wrote the first draft of the manuscript. All authors contributed to the article and approved the submitted version.

## Conflict of Interest

The authors declare that the research was conducted in the absence of any commercial or financial relationships that could be construed as a potential conflict of interest.

## Publisher’s Note

All claims expressed in this article are solely those of the authors and do not necessarily represent those of their affiliated organizations, or those of the publisher, the editors and the reviewers. Any product that may be evaluated in this article, or claim that may be made by its manufacturer, is not guaranteed or endorsed by the publisher.
