# Supplementary material for: Trabecular Bone Microarchitecture Improvement Is Associated With Skeletal Nerve Increase Following Aerobic Exercise Training in Middle-Aged Mice
Source: Front Physiol. 2022 Feb 22;12:800301. doi: 10.3389/fphys.2021.800301 (PMC8902445; doi:10.3389/fphys.2021.800301)
Supplement: Supplementary file 1 [file Data_Sheet_1.docx]

**Supplementary Information**

**Supplementary Figures and Legend**

**Supplementary Figure S1.**

**
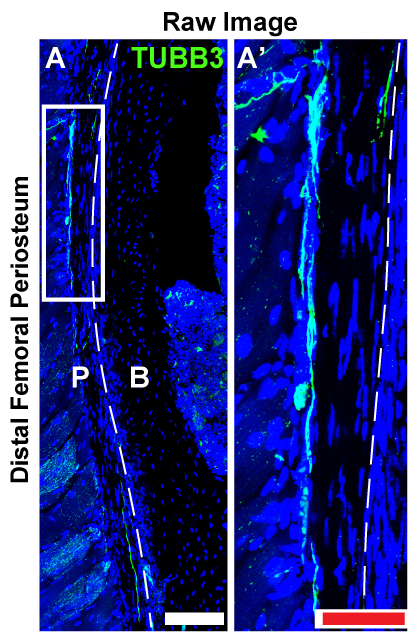
**

**Supplementary Figure S1. Three-dimensional raw images of skeletal nerve fibers in the periosteum.**

Representative raw images of pan-neuronal beta III tubulin (TUBB3) immunohistochemical staining in distal femoral periosteum prior to surface rendering of nerve fibers **(A)**. Higher magnification images were shown **(A’)**. Dashed white line represents a boundary between the periosteum (P) and adjacent cortical bone (B). White scale bar: 100 µm, red scale bar: 50 µm.

**Supplementary Tables**

| **Supplementary Table S1.** Pre- and post-exercise body masses, heart, muscle, and epididymal fat weight of animals following aerobic exercise program. | | | | | | | | | | |
| --- | --- | --- | --- | --- | --- | --- | --- | --- | --- | --- |
| **Weight** | | | | **Non-Exercise** | | | **Aerobic Exercise** | | |  |
| Pre-Ex Body Mass **(g)** | | | | 34.6±1.7 | | | 35.0±1.9 | | |  |
| Post-Ex Body Mass **(g)** | | | | 36.0±1.9 | | | 34.2±2.4 | | |  |
| Heart **(mg)** | | | | 133.2±8.7 | | | 137.5±12.0 | | |  |
| Gastrocnemius **(mg)** | | | | 149.95±8.9 | | | 149.4±9.2 | | |  |
| Plantaris **(mg)** | | | | 19.2±1.3 | | | 19.6±1.8 | | |  |
| Soleus **(mg)** | | | | 8.5±4.1 | | | 11.9±1.9 | | |  |
| Epididymal Fat **(mg)** | | | | 1117.5±219.2 | | | 734.9±293.8 * | | |  |
| **Values represent Means ± SD. *p<0.05** | | | | | | | | | | |
|  |  |  | |  |  | |  |  | | |

| **Supplementary Table S2. Predictive actions of miRNAs over-expressions in bone tissue following aerobic exercise.** | | | | |
| --- | --- | --- | --- | --- |
| **miRNA** | **Expression in Aerobic Exercise** | **Target**  **Organ** | **Action** | **Reference** |
| miR-491-3p | Over-expression | Bone | ↓ with post-menopause-associated osteoporosis  ↑ inhibition of osteoblast apoptosis  ↑ Osteoblast proliferation & differentiation | (Hu et al., 2020) |
|  |  |  |  |  |
| miR-130b-5p | Over-expression | Nerve (brain)  CNS | ↑ expression in brain cells  ↑ neural progenitor proliferation  ↑ Overexpression promote neural progenitor cell proliferation  Upregulation of miR-130b-5p attenuates neuronal cell apoptosis | (Jönsson et al., 2015; Zhang et al., 2018) |
|  |  |  |  |  |
| Let-7a-5p | Over-expression | Schwann cells | ↑ adipose-derived stem cells differentiation into Schwann cells  ↑ the level of Schwann cell related markers (p75NTR, β-NGF, etc.) | (Wang et al., 2021) |
|  |  |  |  |  |
| miR-137-3p | Over-expression | Motoneuron | ↓ Calpain2 level and neuronal nitric oxide synthase (nNOS)  ↓ Motoneuron apoptosis in the spinal cord / promote motoneuron regeneration | (Tang et al., 2018) |
|  |  |  |  |  |
| miR-130a-3p | Over-expression | Dorsal root ganglia (DRG) /  Peripheral Nerve System / Bone | ↑ VEGFR-2 expression in sensory DRG / regulates peripheral nerve system and promotes axonal growth  ↑ osteogenic differentiation of adipose-derived stem cells by increasing Wnt-β catenin signaling | (Glaesel et al., 2020; Yang et al., 2020) |
|  |  |  |  |  |
| miR-29b-3p | Over-expression | Bone (femoral fracture healing) | ↑ osteogenic differentiation of mesenchymal stem cells  ↑ Bone volume during fracture repair and ↑ BMD of the fracture callus | (Lee et al., 2016) |
|  |  |  |  |  |
|  | | | | |

**Predicted roles of miRNAs over-expression in nerve growth and osteogenesis are listed. miR; microRNA, CNS; Central nervous system, NTR; Neurotrophin Receptor, NGF; Nerve growth factor, VEGFR; Vascular growth factor receptor, BMD; Bone mineral density.**

| **Supplementary Table S3. Predictive actions of miRNAs down-expressions in bone tissue following aerobic exercise.** | | | | |
| --- | --- | --- | --- | --- |
| **miRNA** | **Expression in Aerobic Exercise** | **Target**  **Organ** | **Action** | **Reference** |
| miR-3064-5p | Down-expression | Bone (Osteoblast) | miR-3064-5p inhibits osteoblastic differentiation, OCN, OPN, Runx2, and ALP expression /  Knockdown of miR-3064-5p ↑ osteoblast differentiation and osteoblast associated gene expression | (Huang et al., 2021) |
|  |  |  |  |  |
| miR-574-5p | Down-expression | Bone (Osteoclast) | ↑ expression of miR-574-5p induces osteoclastogenesis and promotes osteoclast differentiation | (Hegewald et al., 2020) |
|  |  |  |  |  |
| miR-1187 | Down-expression | Bone (Osteoblast) | ↑ miR-1187 expression inhibits osteoblast differentiation and silencing miR-1187 improves trabecular bone microarchitecture in OVX mice by suppressing BMP2 signaling | (John et al., 2018) |
|  |  |  |  |  |
| miR-154-5p | Down-expression | Bone (Osteoblast) | ↓ miR-154-5p level promotes osteogenic differentiation of adipose-derived stem cells via activating Wnt/PCP signaling pathway | (Li et al., 2015) |
|  |  |  |  |  |
| miR-210-3p | Down-expression | Bone | ↓ miR-210-3p expression inhibits osteogenic, but promotes adipogenic differentiation of MSC via Wnt signaling | (Li et al., 2019a) |
|  |  |  |  |  |
| miR-297a-5p | Down-expression | Bone | ↑ miR-297a-5p inhibits RUNX2 signaling and impairs osteoblastic differentiation of MSC | (Yang et al., 2018) |
|  |  |  |  |  |
| miR-485-3p | Down-expression | Neurons / Neural Stem Cells (NSC) | Knockdown of miR-485-3p expression promotes neuronal viability and reduces neuroinflammation    ↑ miR-485-3p suppresses NSC growth, proliferation, differentiation. Repressing miR-485-3p may promote neuro-regeneration | (Gu et al., 2020; Yu et al., 2021) |
|  |  |  |  |  |
| let-7i-5p | Down-expression | NSC | ↓ let-7i-5p increases NTRK3 expression in NSC and neuronal differentiation | (Cui et al., 2021) |
|  |  |  |  |  |
|  | | | | |

**Predicted roles of miRNAs down-expression in nerve growth and osteogenesis are listed. miR; microRNA, OCN; Osteocalcin, OPN; Osteopontin, ALP; Alkaline phosphatase, OVX; Ovariectomy, BMP2; Bone morphogenic protein 2, MSC; Mesenchymal stem cell, NTRK3; Neurotrophic receptor tyrosine kinase 3 (TrkC).**
